# Changes of bone turnover markers and bone mineral density among postmenopausal Thai women with osteoporosis receiving generic risedronate

**DOI:** 10.1186/s12905-024-03404-5

**Published:** 2024-10-26

**Authors:** Ammarin Suwan, Chotetawan Tanavalee, Krasean Panyakhamlerd, Srihatach Ngarmukos, Suchanant Chavaengkiat, Aree Tanavalee, Chavarin Amarase, Thanapob Bumphenkiatikul

**Affiliations:** 1 Division of Gender, Sexual, and Climacteric Medicine, Department of Obstetrics and Gynecology, Faculty of Medicine, Chulalongkorn University, Bangkok, Thailand; 2 Center of Excellence in Transgender Health (CETH), Faculty of Medicine, Chulalongkorn University, Bangkok, Thailand; 3 Biologics for Knee Osteoarthritis Research Unit, Faculty of Medicine, Chulalongkorn University, Bangkok, Thailand; 4 Department of Orthopaedics, Faculty of Medicine, King Chulalongkorn Memorial Hospital, Chulalongkorn University, Bangkok, Thailand; 5 Department of Academic Affairs, Faculty of Medicine, Chulalongkorn University, Bangkok, Thailand

**Keywords:** Risedronate, Bone turnover markers, Bone mineral density, Osteoporosis, Postmenopausal women

## Abstract

**Background:**

Osteoporosis has been recognized as a significant health issue in Thailand. Pharmacological interventions are important way to prevent fracture. However, one of the main challenges in selecting a medication is high cost, particularly for brand-name drugs. Data on generic bisphosphonate use in Thai are still lacking. Therefore, our study aimed to assess the efficacy and safety of generic risedronate in postmenopausal Thai women with osteoporosis.

**Methods:**

This prospective study was conducted at King Chulalongkorn Memorial Hospital, Bangkok, Thailand, from December 2022 to January 2024. Serum C-terminal cross-linking telopeptide of type I collagen (CTX) and procollagen type I N-propeptide (P1NP) were measured at baseline. All participants subsequently received 35 milligrams of oral risedronate once weekly for 52 weeks. Serum CTX and P1NP were remeasured at different time points. BMD was reevaluated at 52 weeks after risedronate treatment initiation.

**Results:**

A total of 80 participants were included. The mean age was 65.2 ± 6.6 years. The mean body mass index (BMI) was 23.45 ± 3.49 kg/m^2^. The median (IQR) serum CTX level at 12 weeks was significantly lower than that at baseline (0.28 (0.16–0.46) ng/mL versus 0.44 (0.26–0.64) ng/mL, respectively; p value < 0.01). The suppression of serum CTX was confirmed at 52 weeks after treatment initiation. Compared with those at baseline, the serum P1NP levels were significantly lower at 24 weeks after treatment initiation (30.33 (19.19–39.58) ng/mL versus 41.90 (30.33–68.67) ng/mL, respectively; p value < 0.01). In terms of the BMD assessment at 52 weeks, significant improvements were observed in both areal BMD (g/cm^2^) and T scores at all measured sites compared with baseline. The lumbar spine, femoral neck, and total hip BMD increased from baseline by 4.76%, 3.84% and 4.54%, respectively.

**Conclusion:**

Postmenopausal women with osteoporosis who were treated with generic risedronate demonstrated significant suppression of the bone remodelling process at 3, 6, and 12 months after treatment initiation. Additionally, significant improvements in the lumbar spine, femoral neck, and total hip BMD were observed at 12 months of therapy. These findings suggest that generic risedronate could be considered a reasonable and interesting option for treating postmenopausal women with osteoporosis in Thailand.

## Background

Osteoporosis has been recognized as a significant health issue in Thailand [1, 2]. According to a community-based study conducted across multiple regions of Thailand, the prevalence of osteoporosis among women aged 40–80 years is as high as 19.8% at the lumbar spine and 13.6% at the femoral neck. A recent study showed the prevalence of osteoporosis is still high at 21.3%, and patients who present high FRAX scores should have osteoporotic treatment at 10.8%[1]. When age groups were considered, it was observed that older age, particularly an age over 70 years, was correlated with the prevalence of osteoporosis, which was found in half of the study population [1, 3].

Osteoporosis is characterized by low bone mass and the deterioration of bone tissue and is often referred to as a ‘silent disease’ owing to its symptomless progression until the manifestation of fractures [4]. Fracture can profoundly affect quality of life, leading to difficulties in movement, negative impacts on work, loss of work productivity, social lives, a need for daily assistance, depression, and potentially death [5, 6]. Among women who experienced hip fractures, the death rates at 3, 6, and 12 months were 9-17.4%, 12-24.6%, and 17–33%, respectively [7, 8]. Therefore, these findings highlight the importance of early detection, lifestyle modifications and pharmacological interventions [9].

The diagnosis of osteoporosis relies on the criteria established by the World Health Organization (WHO), which involves measuring bone mineral density (BMD) via dual energy X-ray absorptiometry (DXA). Osteoporosis is characterized by a T score of equal to or less than − 2.5 [10]. However, the utilization of DXA is limited by its high cost and limited availability in developing countries such as Thailand.

In terms of bone assessment, intermediate fracture endpoints, such as BMD and bone turnover markers (BTMs) assessment, have been utilized to evaluate bone status. BMD evaluation involves a static test and serves as a standard diagnostic and monitoring tool in osteoporosis treatment and follow-up. However, owing to the slow changes in bone density, significant alterations in BMD should be clinically assessed at least 12 months after the initiation of treatment. In contrast, dynamic tests, such as measurements of BTMs, which reflect bone metabolism, may reveal changes within a shorter period, typically 3 to 6 months after treatment initiation. As a result, BTMs are employed to assess bone changes following treatment, evaluate drug efficacy, and monitor patient compliance.

BTMs are substances generated during bone modelling and remodelling processes, which involve the resorption of old bone by osteoclasts and the formation of new bone by osteoblasts. These markers provide dynamic insights into skeletal status. During bone resorption, osteoclasts degrade collagen and release the C-terminal cross-linking telopeptide of type I collagen (CTX), a nonhelical fragment containing cross-linking regions. This process is mediated by cathepsin K, an osteoclast-specific protease [11–14]. On the other hand, bone formation involves the formation of osteoid, which is primarily composed of type I collagen, during the early phase. Osteoblasts or chondroblasts synthesize procollagen, which undergoes cleavage to form type 1 collagen and the procollagen type I N-propeptide (P1NP) during bone formation. P1NP has three pro α peptides, two α1 chains and one α2 chain. The International Osteoporosis Foundation and the International Federation of Clinical Chemistry and Laboratory Medicine recommend the use of a bone formation marker (serum P1NP) and a bone resorption marker (serum CTX) as reference markers, which are measured via standardized assays in clinical practice [15]. Furthermore, we measured serum BTMs on the basis of the IOF recommendation as early as 3 months after treatment for bone resorption markers and 6 months after treatment for bone formation markers [13, 16].

Thailand was recently classified as a country with an aged society. The incidence of osteoporosis and osteoporotic fractures in the next few decades was predicted. In other words, more patients will need to be treated, and more pharmacological agents will need to be prescribed. Pharmacological interventions for osteoporosis encompass various groups of medications. Bisphosphonates are preferred primarily for treating postmenopausal women with osteoporosis. In Thailand, several types of bisphosphonates are available, including alendronate, risedronate, ibandronate, and zoledronate. However, one of the main challenges in the selection of medications in this group is the high cost, particularly for brand-name drugs, which are more expensive than generic alternatives. Affordable pharmacological agents with proven clinical efficacy should be considered reasonable options. Our study addresses this need by focusing on a Thai cohort, providing data that can inform regional healthcare practices and policies, and adding valuable insights to the field of osteoporosis treatment in resource-limited settings.

## Methods

### Study design

This was a single-centre, prospective trial conducted at King Chulalongkorn Memorial Hospital, Bangkok, Thailand, between December 2022 and January 2024. This study was approved by the Institutional Review Board of the Faculty of Medicine, Chulalongkorn University, Bangkok, Thailand (IRB 115/61), reviewed by the Thai Clinical Trial Registry Committee, and approved for registration and the Thai Clinical Trial Registry (identification number TCTR20240427002) on 27 April 2024. All the enrolled participants received information about the study, and written informed consent was obtained from all participants before the start of the study.

### Inclusion and exclusion criteria

All postmenopausal women with osteoporosis who were aged between 45 and 75 years and visiting the Gender Health Clinic and Orthopaedics Clinic at King Chulalongkorn Memorial Hospital were approached for participation. In our study, postmenopausal women with osteoporosis were defined as those who had not menstruated for at least 1 year, had been diagnosed with osteoporosis by dual-energy X-ray absorptiometry (DXA) and had a T score < -2.5 for at least one of the following sites: the total lumbar spine (or at least 2 out of the 4 lumbar spine (L1-L4) levels), the femoral neck, or the total hip.

The exclusion criteria were postmenopausal women with osteoporosis who had been treated with anti-osteoporotic agents in the past twelve months (i.e., bisphosphonates, hormone therapy, raloxifene, calcitonin, teriparatide, denosumab, abaloparatide, and romosozumab) as well as medications known to interfere with BTMs (such as corticosteroids and antiepileptic drugs). Women who used calcium and vitamin D were allowed to participate in this study. Additionally, women who had experienced recent fractures in the past twelve months were excluded. Furthermore, women with any known conditions affecting BTMs (thyroid disorders, parathyroid disorders, renal insufficiency (GFR < 35 mL/min), rheumatoid disease, Cushing’s disease, multiple myeloma, cancers and diabetes mellitus were also excluded from our study.

Moreover, women who were unable to sit or stand in an upright position for at least 30 min after taking medications and women who experienced severe upper gastrointestinal tract symptoms (such as gastritis, gastroesophageal reflux, oesophageal stenosis, and upper gastrointestinal bleeding) were also excluded.

### Sample size justification

The sample size was calculated using the formula of two dependent means. Values from a previous study was used in the formula, including a standard deviation (SD) of 0.03, a delta of 0.01, an α of 0.05 and a β of 0.2. The calculated sample size in this study was 71 participants. Considering a 20% dropout rate, the total sample size required for the study was 80 participants.

### Data collection and intervention

The demographic data were recorded, and blood samples for serum CTX, P1NP, BUN, creatinine, GFR, calcium, phosphate, albumin, and 25(OH) vitamin D measurement were obtained before generic risedronate (baseline data) was prescribed. Blood was drawn between 8.00 and 9.00 a.m. after the participants fasted overnight for at least 8 h. Serum CTX and P1NP levels were measured via an electrochemiluminescence immunoassay (Elecsys kit; Roche Diagnostics, Thailand). The interassay coefficients of variation (CVs) of serum CTX and P1NP were 3.8% and 2.3%, respectively. The intra-assay CV of serum CTX and P1NP were 2.1% and 1.8%, respectively. All participants subsequently received oral generic risedronate (35 milligrams) weekly for a total of 52 weeks. In participants who had normal serum 25(OH) vitamin D levels, oral vitamin D2 (20,000 IU ergocalciferol) along with 1000 milligrams of oral calcium carbonate once weekly were prescribed. On the other hand, women with low 25(OH) vitamin D levels, defined as those with levels less than 30 ng/mL, received a loading dose of ergocalciferol, followed by repeated serum 25(OH) D measurements. Subsequently, 20,000 IU of ergocalciferol along with 1000 milligrams of calcium carbonate once weekly was prescribed. All participants were advised to sit or stand in an upright position for at least 30 min after taking risedronate to prevent upper gastrointestinal tract irritation.

### Outcome measurements

**Fig. 1:**
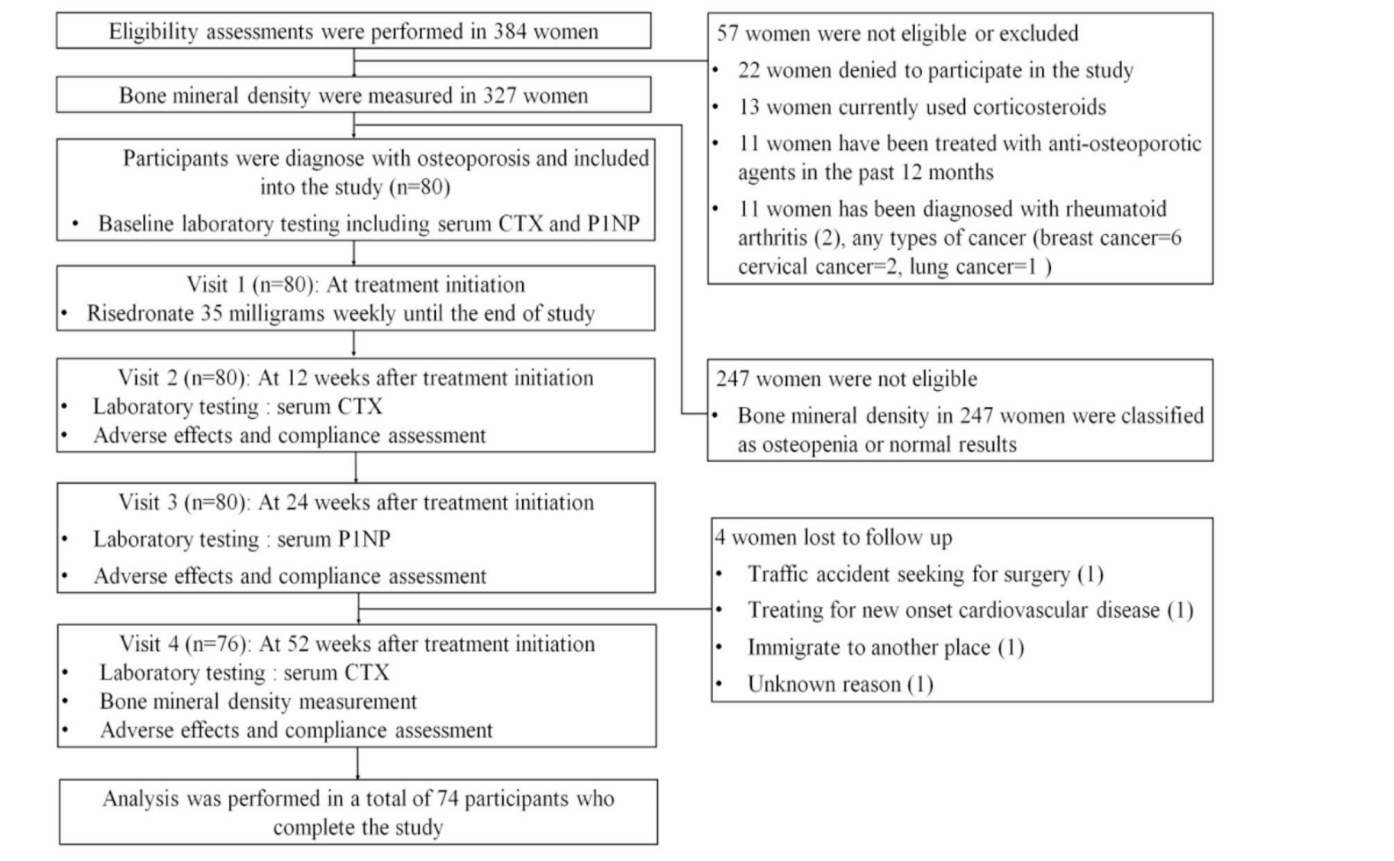
Study flow

The primary outcome was the change in serum CTX levels between baseline and 12 weeks after generic risedronate treatment initiation. The secondary outcomes included the changes in serum CTX levels between baseline and 52 weeks after treatment initiation and serum P1NP levels between baseline and 24 weeks after treatment initiation. Comparisons of the lumbar spine, femoral neck, and total hip BMD at 52 weeks after treatment initiation and at baseline were also performed. Finally, we evaluated the percentages of participants who experienced adverse effects from generic risedronate treatment, such as upper gastrointestinal tract problems (e.g., nausea, vomiting, gastritis, GERD), fever, muscle sprain, and abnormal kidney function. A flowchart of the study is presented in Fig. 1.

### Statistical analysis

IBM SPSS Statistics version 29 was used for the statistical analysis. Descriptive statistics are used to present the baseline data. Comparisons of median serum CTX and P1NP levels at baseline and after generic risedronate treatment were performed using the Wilcoxon signed-rank test. The change in lumbar spine, femoral neck, and total hip BMD between baseline and 52 weeks after treatment initiation was analysed via paired t tests. A p value of < 0.05 was considered statistically significant.

## Results

### Study participants

From December 2022 to January 2024, 384 postmenopausal women were assessed for eligibility. BMD was measured in 327 women. A total of 80 postmenopausal women with osteoporosis met the inclusion criteria and were willing to participate in this study. All participants received 35 milligrams of oral risedronate once weekly. Vitamin D2 along with calcium carbonate was prescribed as mentioned above. Six participants in this study were lost to follow-up between 24 and 52 weeks. The comparative statistical analysis included data from a total of 74 postmenopausal women with osteoporosis who completed the study.

The baseline characteristics of the participants are presented in Table 1. The mean (*±* SD) age of the participants was 65.2 *±* 6.6 years, and the mean age at menopause was 50.2 *±* 4.2 years. The mean body mass index (BMI) was 23.45 ± 3.49 kg/m^2^. None of the participants smoked cigarettes or drank alcohol.

**Table 1:** Baseline characteristics

Variables	Results
Age (years)	65.2±6.60^a^
Age at menopause (years)	50.2±4.20^a^
BMI (kg/m^2^)	23.45±3.49^a^
Serum CTX level (ng/mL)	0.44 (0.26-0.64)
Serum P1NP level (ng/mL)	41.90 (30.33-68.67)
Serum calcium level (ng/mL)	9.60 (9.32-9.90)
Serum phosphorus level (ng/mL)	3.60 (3.30-3.80)
25-OH vitamin D level (ng/mL)	27.10 (21.60-37.30)
Serum creatinine level (mg/dL)	0.65 (0.59-0.75)
Glomerular filtration rate	81.48 (71.94-90.03)

Normally distributed data are presented as the mean ± standard deviation, and data with skewed distributions are presented as the median (IQR). Categorical data are presented as percentages.

BMI: body mass index, CTX: C-terminal cross-linking telopeptide of type I collagen, IQR: interquartile range, P1NP: procollagen type I N-propeptide

Normally distributed data are presented as the mean ± standard deviation, and data with skewed distributions are presented as the median (IQR). Categorical data are presented as percentages.

BMI: body mass index, CTX: C-terminal cross-linking telopeptide of type I collagen, IQR: interquartile range, P1NP: procollagen type I N-propeptide.

In the total study cohort (*n* = 80), there were 111 patients with osteoporosis at any site. While 49 participants were diagnosed with osteoporosis at one site, 31 participants were diagnosed with osteoporosis at two or more sites. The prevalence rates of femoral neck, lumbar, and total hip osteoporosis were 57 cases/111 participants (53.27%), 40 cases/111 participants (37.38%), and 10 cases/111 participants (9.35%), respectively. When the participants were separated into two groups according to age (45–60 years and > 60 years), lumbar osteoporosis was the most prevalent among those aged 45–60 years. The frequencies and percentages of patients with osteoporosis at the lumbar spine, femoral neck, and total hip in this group were 10/20 (50%), 9/20 (45%), and 1/20 (5%), respectively. Conversely, the most common site of osteoporosis in participants aged greater than 60 years was the femoral neck. The frequencies and percentages of patients with osteoporosis at the lumbar spine, femoral neck, and total hip in the latter group were 33/91 (36.26%), 49/91 (53.85%), and 9/91 (9.89%), respectively.

The median (interquartile range, IQR) serum CTX, P1NP, and vitamin D levels at baseline were 0.44 (0.26–0.64) ng/mL, 41.90 (30.33–68.67) ng/mL, and 27.1 (21.60–37.30) ng/mL, respectively. All participants had serum creatinine, calcium, and phosphate levels and glomerular filtration rates (GFRs) at baseline within normal values.

### Comparison of serum bone turnover markers before and after risedronate treatment

As a primary outcome, the change in serum CTX levels between baseline and 12 weeks after risedronate treatment initiation was evaluated. The median (IQR) serum CTX level at 12 weeks was significantly lower compared to baseline (0.28 (0.16–0.46) ng/mL versus 0.44 (0.26–0.64) ng/mL, respectively; p value < 0.01). The suppression of serum CTX was confirmed at 52 weeks after treatment initiation. The median (IQR) serum CTX level at 52 weeks was 0.28 (0.17–0.37) ng/mL (p value < 0.01). The data are presented in Table 2.

**Table 2:** Changes in the levels of serum bone turnover markers after risedronate treatment

Type of bone turnover makers	Serum bone turnover markers at different time points (ng/mL)				Change from baseline			*p* value		
	Baseline	12 weeks	24 weeks	52 weeks	12 weeks	24 weeks	52 weeks	12 weeks	24 weeks	52 weeks
CTX	0.44 (0.26–0.64)	0.28 (0.16–0.46)	-	0.28 (0.17–0.37)	-36.4%	-	-36.4%	< 0.01	-	< 0.01
P1NP	41.90 (30.33–68.67)	-	30.33 (19.19–39.58)	-	-	-27.6%	-	-	< 0.01	-

Data are presented as the median (IQR) and were analysed with the Wilcoxon signed rank test

IQR: interquartile range, CTX: C-terminal cross-linking telopeptide of type I collagen, P1NP: procollagen type I N-propeptide

In terms of bone formation markers, serum P1NP levels were compared between baseline and 24 weeks after treatment initiation. Significantly lower P1NP levels were noted at 24 weeks after treatment initiation compared to baseline (30.33 (19.19–39.58) ng/mL and 41.90 (30.33–68.67) ng/mL, respectively; p value < 0.01). The data are also shown in Table 2.

### Comparison of bone mineral density before and 52 weeks after risedronate treatment initiation

Following 52 weeks of risedronate treatment, significant improvements in both BMD values (g/cm^2^) and T scores across various skeletal sites were observed compared with baseline measurements. In the lumbar spine, the BMD increased from a mean of 0.73 ± 0.10 g/cm^2^ at baseline to 0.77 ± 0.13 g/cm^2^ at 52 weeks, reflecting a substantial increase of 4.76% from baseline (*p* < 0.01). Additionally, the lumbar T score improved from − 2.27 ± 1.04 to -1.91 ± 1.51. Similarly, at the femoral neck, the BMD increased from 0.52 ± 0.07 g/cm^2^ at baseline to 0.54 ± 0.08 g/cm^2^ at 52 weeks, indicating a 3.84% increase from baseline (*p* < 0.01). The femoral neck T score improved from − 2.63 ± 0.73 to -1.89 ± 1.06. At the hip, the BMD increased from 0.66 ± 0.08 g/cm^2^ at baseline to 0.69 ± 0.09 g/cm^2^ at 52 weeks, demonstrating a 4.54% increase from baseline (*p* < 0.01). The total hip T score improved from − 1.66 ± 0.73 to -1.51 ± 0.74. These findings underscore the significant enhancement in bone density across various skeletal sites following generic risedronate treatment, as evidenced by both BMD and T score measurements. The data are presented in Table 3.

**Table 3:** Changes in bone mineral density after risedronate treatment

Parameters		Baseline	52 weeks	Change from baseline	*p* value
Lumbar spine	BMD (g/cm^2^) T score	0.73 *±* 0.10 -2.27 *±* 1.04	0.77 *±* 0.13 -1.91 *±* 0.85	4.76%	< 0.01
Femoral neck	BMD (g/cm^2^) T score	0.52 *±* 0.07 -2.63 *±* 0.73	0.54 *±* 0.08 -1.89 *±* 1.06	3.84%	< 0.01
Total hip	BMD (g/cm^2^) T score	0.66 *±* 0.08 -1.66 *±* 0.73	0.69 *±* 0.09 -1.51 *±* 0.74	4.54%	< 0.01

The data are presented as the means ± SDs and were analysed with paired t tests

BMD: bone mineral density

Following risedronate treatment, possible adverse drug effects were recorded. Four participants (5.00%) experienced new-onset dyspepsia and heartburn. None of the participants required endoscopic or surgical interventions. Three participants (3.75%) experienced back pain, and two participants (2.50%) reported pyrexia with myalgia. Fortunately, pyrexia with myalgia was effectively resolved with symptomatic treatment within three days. Additionally, one participant (1.25%) experienced nausea, and another participant (1.25%) experienced diarrhoea. The data are presented in Table 4.

**Table 4:** Adverse events during the 52-week period

Adverse events	Number of affected participants (%)
New-onset dyspepsia and heartburn	4 (5.00%)
Back pain	3 (3.75%)
Pyrexia with myalgia	2 (2.50%)
Nausea	1 (1.25%)
Diarrhoea	1 (1.25%)

## Discussion

In our study, generic risedronate demonstrated efficacy in osteoporosis treatment, as evidenced by both BTMs and BMD assessments. With respect to BTMs testing, the magnitude of changes in CTX and P1NP levels observed in our study were comparable to those reported in the brand-name risedronate trial [17]. As BTMs are considered integral to bone quality assessments and reflect the mechanism of action of osteoporosis medications, the suppression of the bone remodelling process could imply that bone resorption has been suppressed by this agent. According to the Asian regional guideline consensus, significant changes in serum CTX and P1NP levels are indicated by reductions of more than 30% and 20%, respectively, from baseline [18]. Our study revealed that generic risedronate achieved these goals without causing any serious adverse events.

There are several limitations in interpreting BTMs. Intraindividual variability, interobserver variation, analytic reliability, and poorly and differently defined abnormal cut-off levels are issues of concern regarding the utility of BTMs. Moreover, changes in BTMs levels cannot be relied upon for the diagnosis of osteoporosis. BMD remains the standard method used in both clinical practice and osteoporosis trials. Currently, BTMs are employed primarily for identifying patients with poor responses and nonadherence to therapy and can serve as indicators for restarting bisphosphonate therapy after a drug holiday period [19].

BMD also significantly improved in postmenopausal women with osteoporosis after 12 months of generic risedronate treatment. Significant changes in BMD were detected at all the measured sites, with the most pronounced improvement observed at the lumbar spine. The changes in BMD observed in our study were comparable to those reported in previous studies of brand-name risedronate [17]. All participants in our study experienced no new osteoporotic fractures during the study period. However, owing to the short follow-up period and the absence of a comparator group in our study, the efficacy of fracture reduction has not been confirmed.

Risedronate, as a bisphosphonate, works by inhibiting osteoclast-mediated bone resorption. It binds strongly to hydroxyapatite crystals in bone, which prevents osteoclasts from adhering to the bone surface and reduces their activity. Risedronate specifically inhibits the enzyme farnesyl pyrophosphate synthase within the mevalonate pathway, which is essential for osteoclast function and survival. This inhibition disrupts the formation of important lipids needed for osteoclast function, ultimately leading to decreased bone turnover and improved bone density [12, 20]. This mechanism helps explain the significant reductions in bone turnover markers (BTMs) such as CTX and P1NP observed in our study.

Interestingly, osteoporosis at the femoral neck was more prevalent in postmenopausal women in this study than osteoporosis at the lumbar spine or total hip. This finding is in line with that of our previous study [21] but conflicts with findings from another Asian study [22]. The exact mechanism underlying these differences is currently unknown. However, further studies are still needed to confirm and explain this issue. The possible causes are vertebral endplate sclerosis and osteoarthritis of the facet joint.

A cost-effectiveness analysis of screening and treatment strategies for postmenopausal women with osteoporosis in Thailand revealed that primary prevention had a much lower cost per quality-adjusted life years (QALYs) than did secondary prevention [23]. Among the different treatment options for osteoporosis, risedronate has the lowest cost, followed by alendronate, raloxifene, and nasal calcitonin. However, alendronate has the lowest incremental cost-effectiveness ratio (ICER) for both primary and secondary prevention [23].

In Thailand, a few generic bisphosphonate agents are available, including alendronate and risedronate. Compared with risedronate, alendronate has greater bone affinity but lower potency for inhibiting farnesyl pyrophosphate synthase (FFP). Differences in R1 and R2 structures among bisphosphonate molecules result in different drug structures and properties. Some studies have suggested that risedronate has a quicker onset of anti-fracture action because of its greater FFP inhibition. On the other hand, washout of risedronate from bone might occur more rapidly than that of alendronate because of its lower bone affinity [24, 25]. In clinical practice, the onset of anti-fracture efficacy, the duration of drug accumulation in bone, cost, the availability of drugs, and physician and patient preferences play a role in the selection of drugs for treating postmenopausal women with osteoporosis. In terms of efficacy, the response after risedronate use in our study was confirmed by both BTMs and BMD after 12 months of treatment. However, there is a plateau effect after using any kind of bisphosphonates for 3–5 years. In terms of safety, long-term use of bisphosphonates can increase the risk of osteonecrosis of the jaw and atypical femoral fractures. Therefore, it is necessary to reassess fracture risk (i.e. BMD, FRAX score and/or BTMs) after 3–5 years of bisphosphonate use and evaluate whether to continue treatment or consider a drug holiday [26, 27].

Thailand is a developing country facing economic challenges, including slow economic growth rates that have persisted for decades. Drugs at affordable prices that have been shown to be effective should be considered as part of the country’s health care policy. Many physicians have various medication options for treating osteoporosis, including alendronate, risedronate, zoledronic acid, ibandronate, raloxifene, denosumab, and romosozumab. Moreover, menopausal hormone therapy (MHT) can also be used for Thai postmenopausal women who are younger than 60 years or those with at time since the final menstrual period of less than 10 years. The concept of MHT use for treating postmenopausal women with osteoporosis is primarily based on the International Menopause Society (IMS) recommendations [28]. The effects of MHT on BTMs suppression have been confirmed in many countries, including Thailand. One study revealed that bone protection with BTMs suppression occurred at 3 months after oestrogen treatment [29, 30]. However, contrary to these findings, the American Association of Clinical Endocrinologists/American College of Endocrinology Clinical Practice Guidelines (AACE/ACE) for the Diagnosis and Treatment of Postmenopausal Women with Osteoporosis 2020 stated that oestrogen was never explicitly approved for the treatment of postmenopausal women with osteoporosis [31].

In Thailand, both brand-name and generic forms of risedronate are available in the health care system. The price of generic risedronate is approximately 50% lower than that of the brand-name agent. From an economic standpoint, a generic agent with efficacy proven through intermediate outcome measurements could be considered a reasonable and intriguing option. However, our study was not aimed primarily at evaluating the cost-effectiveness of generic risedronate. Long-term data on quality of life and other important economic data were not collected. Hence, further studies are needed to conduct cost-effectiveness analyses of all available treatment options for osteoporosis patients in Thailand. Another potential benefit of generic risedronate over brand-name risedronate is the increased accessibility of medical intervention for patients. In cases where osteoporosis patients face economic difficulties, the use of generic drugs offers a more accessible and valuable option than the absence of treatment.

In terms of safety and adverse effects, the most prevalent adverse effect reported in our study was new-onset dyspepsia and heartburn. However, the prevalence rates of dyspepsia and heartburn were lower in our study than those reported in the pooled analysis [32]. Additionally, the incidence of adverse effects reported in that study did not significantly differ from that reported in the placebo group [32].

Our study had several limitations. The lack of a control group is one of the major limitations, which reduces the strength of the study’s conclusions, particularly in terms of comparing the efficacy of generic versus branded risedronate. Moreover, given the short follow-up period, the number of participants is another concern affecting the interpretation of our results. Further well-controlled, larger sample size and long-term studies are needed to better understand the long-term effects and safety of risedronate use. The optimal duration of treatment and the timing for reassessing fracture risk or considering a drug holiday should always be considered.

## Conclusion

Postmenopausal women with osteoporosis who were treated with generic risedronate demonstrated significant suppression of the bone remodelling process at 3, 6, and 12 months after treatment initiation. Additionally, significant improvements in the lumbar spine, femoral neck, and total hip BMD were observed after 12 months of therapy. These findings suggest that generic risedronate could be considered a reasonable and interesting option for treating postmenopausal women with osteoporosis in Thailand.

## Data Availability

The datasets used and analysed during the current study are available from the corresponding author upon reasonable request.

## Abbreviations

BMD: Bone mineral density
DXA: Dual energy X-ray Absorptiometry
BTMs: Bone Turnover Markers
CTX: C-terminal cross-linking Telopeptide of type I collagen
P1NP: Procollagen Type I N-Propeptide

## References

[1] Chanidkul P, Sribenjalak D, Charoenngam N, Pongchaiyakul C. The proportion of Thai postmenopausal women who would be eligible for anti-osteoporosis therapy. PLoS ONE. 2023;18:e0279829. 36735672 10.1371/journal.pone.0279829 PMC9897565

[2] Pongchaiyakul C, Songpattanasilp T, Taechakraichana N. Burden of osteoporosis in Thailand. J Med Assoc Thai. 2008;91:261–7. 18389994

[3] Limpaphayom KK, Taechakraichana N, Jaisamrarn U, Bunyavejchevin S, Chaikittisilpa S, Poshyachinda M, Taechamahachai C, Havanond P, Onthuam Y, Lumbiganon P, Kamolratanakul P. Bone mineral density of lumbar spine and proximal femur in normal Thai women. J Med Assoc Thai. 2000;83:725–31. 10932505

[4] Cosman F, de Beur SJ, LeBoff MS, Lewiecki EM, Tanner B, Randall S, Lindsay R. National osteoporosis F: Clinician’s guide to Prevention and treatment of osteoporosis. Osteoporos Int. 2014;25:2359–81. 25182228 10.1007/s00198-014-2794-2 PMC4176573

[5] Suriyawongpaisal P, Chariyalertsak S, Wanvarie S. Quality of life and functional status of patients with hip fractures in Thailand. Southeast Asian J Trop Med Public Health. 2003;34:427–32. 12971576

[6] Yeh EJ, Rajkovic-Hooley O, Silvey M, Ambler WS, Milligan G, Pinedo-Villanueva R, Harvey NC, Moayyeri A. Impact of fragility fractures on activities of daily living and productivity in community-dwelling women: a multi-national study. Osteoporos Int. 2023;34:1751–62. 37335332 10.1007/s00198-023-06822-7 PMC10511617

[7] Vaseenon T, Luevitoonvechkij S, Wongtriratanachai P, Rojanasthien S. Long-term mortality after osteoporotic hip fracture in Chiang Mai, Thailand. J Clin Densitom. 2010;13:63–7. 20171568 10.1016/j.jocd.2009.10.003

[8] Guzon-Illescas O, Perez Fernandez E, Crespi Villarias N, Quiros Donate FJ, Pena M, Alonso-Blas C, Garcia-Vadillo A, Mazzucchelli R. Mortality after osteoporotic hip fracture: incidence, trends, and associated factors. J Orthop Surg Res. 2019;14:203. 31272470 10.1186/s13018-019-1226-6 PMC6610901

[9] LeBoff MS, Greenspan SL, Insogna KL, Lewiecki EM, Saag KG, Singer AJ, Siris ES. The clinician’s guide to prevention and treatment of osteoporosis. Osteoporos Int. 2022;33:2049–102. 35478046 10.1007/s00198-021-05900-y PMC9546973

[10] Kanis JA. Assessment of fracture risk and its application to screening for postmenopausal osteoporosis: synopsis of a WHO report. WHO Study Group. Osteoporos Int. 1994;4:368–81. 7696835 10.1007/BF01622200

[11] Naylor K, Eastell R. Bone turnover markers: use in osteoporosis. Nat Rev Rheumatol. 2012;8:379–89. 22664836 10.1038/nrrheum.2012.86

[12] Eastell R, Szulc P. Use of bone turnover markers in postmenopausal osteoporosis. Lancet Diabetes Endocrinol. 2017;5:908–23. 28689768 10.1016/S2213-8587(17)30184-5

[13] Schini M, Vilaca T, Gossiel F, Salam S, Eastell R. Bone turnover markers: Basic Biology to Clinical Applications. Endocr Rev. 2023;44:417–73. 36510335 10.1210/endrev/bnac031 PMC10166271

[14] Langdahl B, Ferrari S, Dempster DW. Bone modeling and remodeling: potential as therapeutic targets for the treatment of osteoporosis. Ther Adv Musculoskelet Dis. 2016;8:225–35. 28255336 10.1177/1759720X16670154 PMC5322859

[15] Vasikaran S, Cooper C, Eastell R, Griesmacher A, Morris HA, Trenti T, Kanis JA. International Osteoporosis Foundation and International Federation of Clinical Chemistry and Laboratory Medicine position on bone marker standards in osteoporosis. Clin Chem Lab Med. 2011;49:1271–4. 21605012 10.1515/CCLM.2011.602

[16] Delmas PD, Eastell R, Garnero P, Seibel MJ, Stepan J, Committee of Scientific Advisors of the International Osteoporosis F. The use of biochemical markers of bone turnover in osteoporosis. Committee of Scientific Advisors of the International Osteoporosis Foundation. Osteoporos Int. 2000;11(Suppl 6):S2–17. 11193237 10.1007/s001980070002

[17] Watts NB, Chines A, Olszynski WP, McKeever CD, McClung MR, Zhou X, Grauer A. Fracture risk remains reduced one year after discontinuation of risedronate. Osteoporos Int. 2008;19:365–72. 17938986 10.1007/s00198-007-0460-7

[18] Wu CH, Chang YF, Chen CH, Lewiecki EM, Wuster C, Reid I, Tsai KS, Matsumoto T, Mercado-Asis LB, Chan DC, et al. Consensus Statement on the use of bone turnover markers for short-term monitoring of osteoporosis treatment in the Asia-Pacific Region. J Clin Densitom. 2021;24:3–13. 31010789 10.1016/j.jocd.2019.03.004

[19] Camacho PM, Petak SM, Binkley N, Diab DL, Eldeiry LS, Farooki A, Harris ST, Hurley DL, Kelly J, Lewiecki EM, et al. American Association of Clinical Endocrinologists/American College of Endocrinology Clinical Practice Guidelines for the diagnosis and treatment of postmenopausal Osteoporosis-2020 Update. Endocr Pract. 2020;26:1–46. 32427503 10.4158/GL-2020-0524SUPPL

[20] Drake MT, Clarke BL, Khosla S. Bisphosphonates: mechanism of action and role in clinical practice. Mayo Clin Proc. 2008;83:1032–45. 18775204 10.4065/83.9.1032 PMC2667901

[21] Suwan A, Panyakhamlerd K, Chaikittisilpa S, Jaisamrarn U, Hawanond P, Chaiwatanarat T, Tepmongkol S, Chansue E, Taechakraichana N. Validation of the Thai osteoporosis foundation and royal college of orthopaedic surgeons of Thailand clinical practice guideline for bone mineral density measurement in postmenopausal women. Osteoporos Sarcopenia. 2015;1:103–8.

[22] Zeng Q, Li N, Wang Q, Feng J, Sun D, Zhang Q, Huang J, Wen Q, Hu R, Wang L, et al. The prevalence of osteoporosis in China, a Nationwide, Multicenter DXA Survey. J Bone Min Res. 2019;34:1789–97. 10.1002/jbmr.3757 31067339

[23] Kingkaew P, Maleewong U, Ngarmukos C, Teerawattananon Y. Evidence to inform decision makers in Thailand: a cost-effectiveness analysis of screening and treatment strategies for postmenopausal osteoporosis. Value Health. 2012;15:S20–28. 22265062 10.1016/j.jval.2011.11.015

[24] Watts NB, Diab DL. Long-term use of bisphosphonates in osteoporosis. J Clin Endocrinol Metab. 2010;95:1555–65. 20173017 10.1210/jc.2009-1947

[25] Wells GA, Hsieh SC, Zheng C, Peterson J, Tugwell P, Liu W. Risedronate for the primary and secondary prevention of osteoporotic fractures in postmenopausal women. Cochrane Database Syst Rev. 2022;5:CD004523. 35502787 10.1002/14651858.CD004523.pub4 PMC9062986

[26] Kong SY, Kim DY, Han EJ, Park SY, Yim CH, Kim SH, Yoon HK. Effects of a ‘drug holiday’ on bone mineral density and bone turnover marker during bisphosphonate therapy. J Bone Metab. 2013;20:31–5. 24524053 10.11005/jbm.2013.20.1.31 PMC3780827

[27] Liel Y, Plakht Y, Tailakh MA, BONE TURNOVER IN OSTEOPOROTIC WOMEN DURING LONG-TERM ORAL BISPHOSPHONATES TREATMENT. IMPLICATIONS FOR TREATMENT FAILURE AND DRUG HOLIDAY IN THE REAL WORLD. Endocr Pract. 2017;23:787–93. 28448762 10.4158/EP171781.OR

[28] Baber RJ, Panay N, Fenton A, Group IMSW. 2016 IMS recommendations on women’s midlife health and menopause hormone therapy. Climacteric. 2016;19:109–50. 26872610 10.3109/13697137.2015.1129166

[29] Vatrasresth J, Suwan A, Panyakhamlerd K. Effects of early estradiol valerate administration on bone turnover markers in surgically induced menopausal women. BMC Womens Health. 2021;21:363. 34645447 10.1186/s12905-021-01508-w PMC8515676

[30] Dunn CJ, Goa KL. Risedronate: a review of its pharmacological properties and clinical use in resorptive bone disease. Drugs. 2001;61:685–712. 11368289 10.2165/00003495-200161050-00013

[31] Watts NB, Camacho PM, Lewiecki EM, Petak SM, Force AAPOGT. American Association of Clinical Endocrinologists/American College of Endocrinology Clinical Practice Guidelines for the diagnosis and treatment of postmenopausal Osteoporosis-2020 Update. Endocr Pract. 2021;27:379–80. 33577971 10.1016/j.eprac.2021.02.001

[32] Taggart H, Bolognese MA, Lindsay R, Ettinger MP, Mulder H, Josse RG, Roberts A, Zippel H, Adami S, Ernst TF, Stevens KP. Upper gastrointestinal tract safety of risedronate: a pooled analysis of 9 clinical trials. Mayo Clin Proc. 2002;77:262–70. 11888030 10.4065/77.3.262

